# Genetic Diversity and Selection Signatures in Synthetic-Derived Wheats and Modern Spring Wheat

**DOI:** 10.3389/fpls.2022.877496

**Published:** 2022-07-12

**Authors:** Mohsin Ali, Shan Danting, Jiankang Wang, Hafsa Sadiq, Awais Rasheed, Zhonghu He, Huihui Li

**Affiliations:** ^1^Institute of Crop Sciences and CIMMYT China Office, Chinese Academy of Agricultural Sciences (CAAS), Beijing, China; ^2^Nanfan Research Institute, Chinese Academy of Agricultural Sciences (CAAS), Sanya, China; ^3^Department of Plant Sciences, Quaid-i-Azam University, Islamabad, Pakistan

**Keywords:** bread wheat, genotyping-by-sequencing, genetic diversity, EigenGWAS, selection signatures, gene annotation

## Abstract

Synthetic hexaploid wheats and their derived advanced lines were subject to empirical selection in developing genetically superior cultivars. To investigate genetic diversity, patterns of nucleotide diversity, population structure, and selection signatures during wheat breeding, we tested 422 wheat accessions, including 145 synthetic-derived wheats, 128 spring wheat cultivars, and 149 advanced breeding lines from Pakistan. A total of 18,589 high-quality GBS-SNPs were identified that were distributed across the A (40%), B (49%), and D (11%) genomes. Values of population diversity parameters were estimated across chromosomes and genomes. Genome-wide average values of genetic diversity and polymorphic information content were estimated to be 0.30 and 0.25, respectively. Neighbor-joining (NJ) tree, principal component analysis (PCA), and kinship analyses revealed that synthetic-derived wheats and advanced breeding lines were genetically diverse. The 422 accessions were not separated into distinct groups by NJ analysis and confirmed using the PCA. This conclusion was validated with both relative kinship and Rogers' genetic distance analyses. EigenGWAS analysis revealed that 32 unique genome regions had undergone selection. We found that 50% of the selected regions were located in the B-genome, 29% in the D-genome, and 21% in the A-genome. Previously known functional genes or QTL were found within the selection regions associated with phenology-related traits such as vernalization, adaptability, disease resistance, and yield-related traits. The selection signatures identified in the present investigation will be useful for understanding the targets of modern wheat breeding in Pakistan.

## Introduction

Bread wheat (*Triticum aestivum*, also called common wheat) is one of the most important staple cereal crops that feed more than 35% of the world's population (Paux et al., 2008). Bread wheat is an allohexaploid species (2n = 6x = 42, AABBDD genomes) that arose ~8,000-10,000 years ago in Fertile Crescent (Kihara, 1944; McFadden, 1944; McFadden and Sear, 1946) by hybridizations between the tetraploid emmer wheat (*Triticum turgidum*, 2n = 4x = 28; AABB) and diploid wild goatgrass (*Aegilops tauschii*, 2n = 14; DD). Its large and complex hexaploid genome of approximate 16 Gigabases, had hindered genomic analysis in this species (Chapman et al., 2015; Appels et al., 2018). Genomic variations in wheat are mainly driven by multiple factors such as polyploidization events, domestication, spread to new geographical regions from origin sites, gene flow, and post-domestication selection or breeding (Tanno and Willcox, 2006; Luo et al., 2007; Cavanagh et al., 2013; Choulet et al., 2014; Zhou et al., 2018).

Approximately seven decades ago, semi-dwarf spring wheat varieties from Mexico resulted in a breakthrough in Pakistan, India, and other parts of the world (Dowswell, 1989). In subsequent years, improvements in agronomic practices and conventional breeding methods have contributed to a radical increase in cereal crop production, including wheat, which played a crucial role in food security. Consequently, crop improvement activities might have resulted in the loss of genetic diversity (Reif et al., 2005), which could be due to a founder effect associated with a restricted ancestral base. It is now estimated that further increase in the harvest index needs innovations in breeding and germplasm resources. Wild relatives of wheat offer great potential to increase allelic diversity for multiple traits, including grain yield, nutritional quality, and adaptability in stressed environments (Rasheed et al., 2018). Synthetic hexaploid wheats are one of the proven resources to restore lost genetic diversity and introduce untapped genetic variations in elite germplasm (Rasheed et al., 2018). Synthetic hexaploid wheats and their advanced derivatives developed in Pakistan have been reported to have better agronomic performance than non-synthetic wheat (Afzal et al., 2017, 2019). Afzal et al. (2019) evaluated the genetic diversity and population structure of 171 synthetic hexaploid derivatives and 69 bread wheat cultivars from Pakistan using 90 K SNP array. They reported that synthetic derivatives have noticeable differences from bread wheat for genetic diversity patterns, genetic population structure, and haplotype blocks. Also, synthetic derivatives were more genetically diverse as compared to bread wheat cultivars. In another study, a diversity panel comprised of 213 accessions, including synthetic-derived wheats and elite bread wheat cultivars was evaluated using allelic variations of 87 functional genes (Khalid et al., 2019). They observed that synthetic derivatives and bread wheat lines could be separated into two groups. To date, many synthetic derivatives have been used as potential parents to improve the agronomic characteristics of elite cultivars.

During the process of domestication, natural selection, and human-mediated selection, crops have experienced intensive selection for better yield, quality, stress, adaptation, and stress resistance (Yamasaki et al., 2007; Cavanagh et al., 2013; He et al., 2019). Molecular evidence of selection remains in the patterns of genetic variations and selected regions within cultivated genomes. Genes and/or genetic variations within selected regions are always associated with agriculturally important traits and reflect the main driving forces for genome-wide divergence at the population level (Cavanagh et al., 2013; Zhou et al., 2018). In addition, Hyten et al. (2006) indicated that a relatively small number of loci impose phenotypic improvement on modern cultivars in wheat breeding while a large proportion of the genome remains unchanged. Thus, insights into genetic variations and identification of loci under selection during crop improvement can provide valuable guidelines, opportunities, and breeding targets for future breeding programs (Morrell et al., 2012; Cavanagh et al., 2013). Although classical forward genetics approaches (e.g., linkage mapping and genome-wide association mapping) effectively detect causal variants related to specific traits, they are limited to detecting genetic variations associated with domestication and improvement (Morrell et al., 2012; Ramey et al., 2013).

In population genetics, eigenvectors derived from genetic data have extensively been used to quantify the genetic differences across populations and to infer evolutionary history (Patterson et al., 2006; Reich et al., 2009). By combining the statistical framework of genome-wide association studies (GWAS) with eigenvector decomposition, Chen et al. (2016) proposed a method called EigenGWAS (genome-wide association study with eigenvector decomposition), which identifies loci under selection without a requirement for discrete populations (Chen et al., 2016; Li J. et al., 2019). Conceptually, the EigenGWAS statistical framework is similar to conventional GWAS methods, except that the phenotype is substituted with PCA's eigenvector to capture cryptic relationships of the studied population. EigenGWAS has been successfully deployed to identify genomic regions that had undergone selection in recent studies on humans (Chen et al., 2016), pig (Tang et al., 2020), chicken (Zhao et al., 2018), bird (Bosse et al., 2017), insect (You et al., 2020), wheat (Afzal et al., 2019; Liu et al., 2019), maize (Li J. et al., 2019, 2020), rice (Ma et al., 2016), and barley (Li Z. et al., 2020). These studies have identified genomic regions under selection enriched for genes associated with biologically important traits.

To date, a few studies have reported the patterns of genomic variations and identification of genomic loci that had undergone selection in Pakistan wheat germplasm (Afzal et al., 2019; Liu et al., 2019). Therefore, the impact of selective breeding on genomic variations and selection signatures remained poorly understood in Pakistan wheat breeding germplasm. In this study, we used a panel of 422 wheat accessions, including cultivars, advanced lines, and synthetic-derived wheats using GBS technology. Our objectives in this study were to (1) investigate the genetic diversity and population structure of this panel; (2) identify the genomic regions that were directionally selected, and (3) associate the selective regions with reported QTL/gene known to influence traits of breeding interest.

## Materials and Methods

### Plant Materials

A panel of 422 hexaploid wheat accessions was examined for molecular characterization analyses. Seed samples were obtained from the Plant Genetic Resources Institute, National Agricultural Research Center, Islamabad, Pakistan. Based on the given samples information, the 422 wheat accessions (hereafter referred to as the whole population, WP) were classified into three subpopulations, including 145 synthetic-derived wheats (SYN-DER, developed by crossing primary synthetic hexaploid wheats with advanced lines and elite cultivars of Pakistan and CIMMYT), 128 commercially released Pakistan cultivars (PC, i.e., genotypes that are unique and stable, and have been selected for agronomic traits), and 149 advanced lines (AL, i.e., group of lines developed for better agronomic characteristics). Detailed information on the 422 wheat accessions can be found in Supplementary Table S1.

### Genotyping and Quality Control

Five viable seeds of each accession tested in this study were planted in 5 cm diameter plots. Genomic DNA was isolated and purified from fresh leaf samples of 2-week-old seedlings using the cetyltrimethyl ammonium bromide (CTAB) method (Doyle and Doyle, 1987). DNA of all the samples was sent to the Cornell University Biotechnology Resource Center for GBS genotyping. The GBS method was performed according to the protocol proposed by Poland et al. (2012) using a two-enzyme (*MspI-PstI*) approach. A GBS analysis pipeline in TASSEL version 5.0 (Trait Analysis by aSSociation Evolution and Linkage) was used for SNP calling (Bradbury et al., 2007). A total of 133,738,39 GBS-SNPs were retrieved based on the “Chinese Spring” reference genome v.1.0 from International Wheat Genome Sequencing Consortium (IWGSC). Identified GBS-SNPs were named as “chromosome number_physical position, that is, 1A_555961328. More than 50% of the GBS-SNPs were removed from the dataset due to their missing rate being higher than 50%. Unmapped GBS-SNPs were also excluded from the dataset. The remaining SNPs were imputed using Beagle version 5.1 with default parameters (Browning et al., 2018). Then, 18,589 GBS-SNPs with heterozygosity <0.2 and minor allele frequency (MAF) exceeding 0.05 were retained using TASSEL version 5.0 (Bradbury et al., 2007) for the follow-up analysis.

### Population Genomic Parameter Analyses

PowerMarker version 3.25 software (Liu and Muse, 2005) was used to calculate population genomics parameters, including MAF, heterozygosity (He), genetic diversity (GD), and polymorphism information content (PIC) for the WP and each of the three predefined subpopulations (i.e., SYN-DER, PC, and AL). To investigate the patterns of nucleotide variations, transition (Ts) to transversion (Tv) mutation statistics, Tajima's D tests, and nucleotide diversity (π) were evaluated using VCFtools version 0.1.15 (Danecek et al., 2011). The population structure of the WP was assessed using NJ-tree and PCA. NJ analysis was conducted using TASSEL version 5.0, while PCA analysis was performed using an R “SNPrelate” package (Zheng et al., 2012). TASSEL version 5.0 (Bradbury et al., 2007) was used to perform linkage disequilibrium (LD) among pairs of SNPs of each subpopulation by estimating squared allele frequency correlation (r^2^) of alleles. The LD decays within WP and three subpopulations were evaluated, as was the distance among pairs of SNPs with non-linear regression using a custom R script.

The relative kinship analysis implemented in the GAPIT (Genomic Association and Prediction Integrated Tool) R package (Lipka et al., 2012) was performed to reveal the genetic identity (or genetic relationship) between any two given accessions. Negative kinship coefficient values between two accessions, indicating the existence of a weaker genetic relationship than would be expected between two random accessions, were set to zero. Roger's genetic distance was estimated using BIO-R software version 2.0 (Pacheco et al., 2016). Negative genetic distance values were replaced by zero. The analysis of molecular variance (AMOVA) and pairwise F_ST_ analyses were performed using Arlequin 3.5 software to estimate genetic differences between predefined subpopulations (Excoffier et al., 2005).

### Identification of Genomic Regions Under Selection

EigenGWAS implemented in the GEAR software (freely available from https://github.com/gc5k/GEAR), was used to identify genes/QTL that underlying population genetic differences and to detect candidate regions of the wheat genome under selection in any genetic population (Chen et al., 2016). The EigenGWAS is a single marker regression method based on the PCA. It is similar to a typical GWAS method; however, the phenotype is replaced with an individual-level eigenvector (EV) derived from the genotypic data. Briefly, EigenGWAS involved three steps: first, 18,589 high-quality GBS-SNPs were used to generate the genomic relationship matrix for WP; second, the first 10 eigenvalues and their corresponding eigenvectors (i.e., EV1-EV10) were calculated; and third, marker effects were estimated by regressing each GBS-SNP for a selected eigenvector from the second step. More detailed instructions can be found on the “GEAR” software website (https://github.com/gc5k/GEAR/wiki/EigenGWAS). To exclude the effect of genetic drift (Devlin and Roeder, 1999), the *p* was adjusted using a genomic control factor (λ_*GC*_), denoted as *P*_*GC*_, and was used to identify genomic regions under selection. To determine the threshold of significance of genomic regions under directional selection, the first EV was reshuffled 1,000 times to simulate the null distribution. The 95th quantile of the 1,000 most significant *P*_*GC*_ was calculated using 1,000 permutations to determine the cutoff. After log_10_ (*p*) transformation, 5.0 was applied for -log_10_(*P*_*GC*_) of EigenGWAS analyses in all 10 EVs to declare as regions under selection.

### Genome Annotation and Reported QTL/Gene Overlapping With Potential Selected Regions

To exclude the strong effect of LD, significant SNP loci within 5 Mb both up- and downstream based on the LD level of the WP were merged as potential selected regions. Functional annotations of the target GBS-SNPs were performed using SnpEff software (Cingolani et al., 2012). The wheat IWGSC RefSeq Annotation v1.0 as a “ggf3” file format was downloaded from the EnsemblPlants database at https://plants.ensembl.org/. The PANTHER Overrepresentation Test (release 20210224) using Gene Ontology (GO) database (release 2021/05/01) using *Triticum aestivum* database as a reference list. GO analysis included biological process, molecular function, and cellular component. The raw *p* < 0.01 was set as the threshold to declare the significant differential expression.

## Results

### Marker Density and Genetic Diversity

A total of 18,589 high-quality GBS-SNPs were well distributed across the genome in the current diversity panel (Table 1). The GBS-SNPs covered a physical distance of 14,053.03 megabase (Mb), with an average density of 1.26 Mb per SNP. The number of GBS-SNPs identified were 7,423 (40%), 9,035 (49%), and 2,131 (11%) in A, B, and D genomes, respectively (Table 1). Among genomes, the highest and lowest number of GBS-SNPs were recorded on chromosomes 2B (1575 SNPs) and 4D (128 SNPs), respectively. The marker density for the D-genome (0.53 Mb per SNPs) was lower than that for the A- (1.51) and B- (1.74) genomes (Table 1). Chromosome-wise marker density varied from 0.25 (4D) to 2.05 (7A and 7B). Chromosome sizes ranged from 473.05 Mb (6D) to 829.74 Mb (3B).

**Table 1:** The summary statistics of GBS-SNPs across chromosomes and genomes.

**Chromosome**	**No. of markers**	**%SNP**	**Start position**	**End position**	**Length (Mb)**	**Density (Mb/marker)**
						
1A	909	5%	1145442	593501692	593.50	1.53
1B	1,100	6%	1430915	688327586	688.33	1.60
1D	313	2%	78777	493978993	493.98	0.63
2A	1,186	6%	626007	780652409	780.65	1.52
2B	1,575	8%	19097	800780364	800.78	1.97
2D	317	2%	2593153	649073688	649.07	0.49
3A	966	5%	607725	750500626	750.50	1.29
3B	1,355	7%	198860	829742765	829.74	1.63
3D	429	2%	344069	615061869	615.06	0.70
4A	1,003	5%	2013324	743825197	743.83	1.35
4B	723	4%	586028	673071478	673.07	1.07
4D	128	1%	1187858	509798252	509.80	0.25
5A	903	5%	1213423	709755448	709.76	1.27
5B	1304	7%	218401	712940770	712.94	1.83
5D	196	1%	2214591	564899608	564.90	0.35
6A	944	5%	684328	617838760	617.84	1.53
6B	1,438	8%	195536	720519123	720.52	2.00
6D	226	1%	70342	473049509	473.05	0.48
7A	1,512	8%	289461	736572283	736.57	2.05
7B	1,540	8%	88786	750602636	750.60	2.05
7D	522	3%	1570012	638541382	638.54	0.82
A	7,423	40%	289461	780652409	4,932.65	1.51
B	9,035	49%	19097	829742765	5,175.98	1.74
D	2,131	11%	70342	649073688	3,944.40	0.53
The whole genome	18,589	100%	19097	829742765	14,053.03	1.26

**Figure 1:**
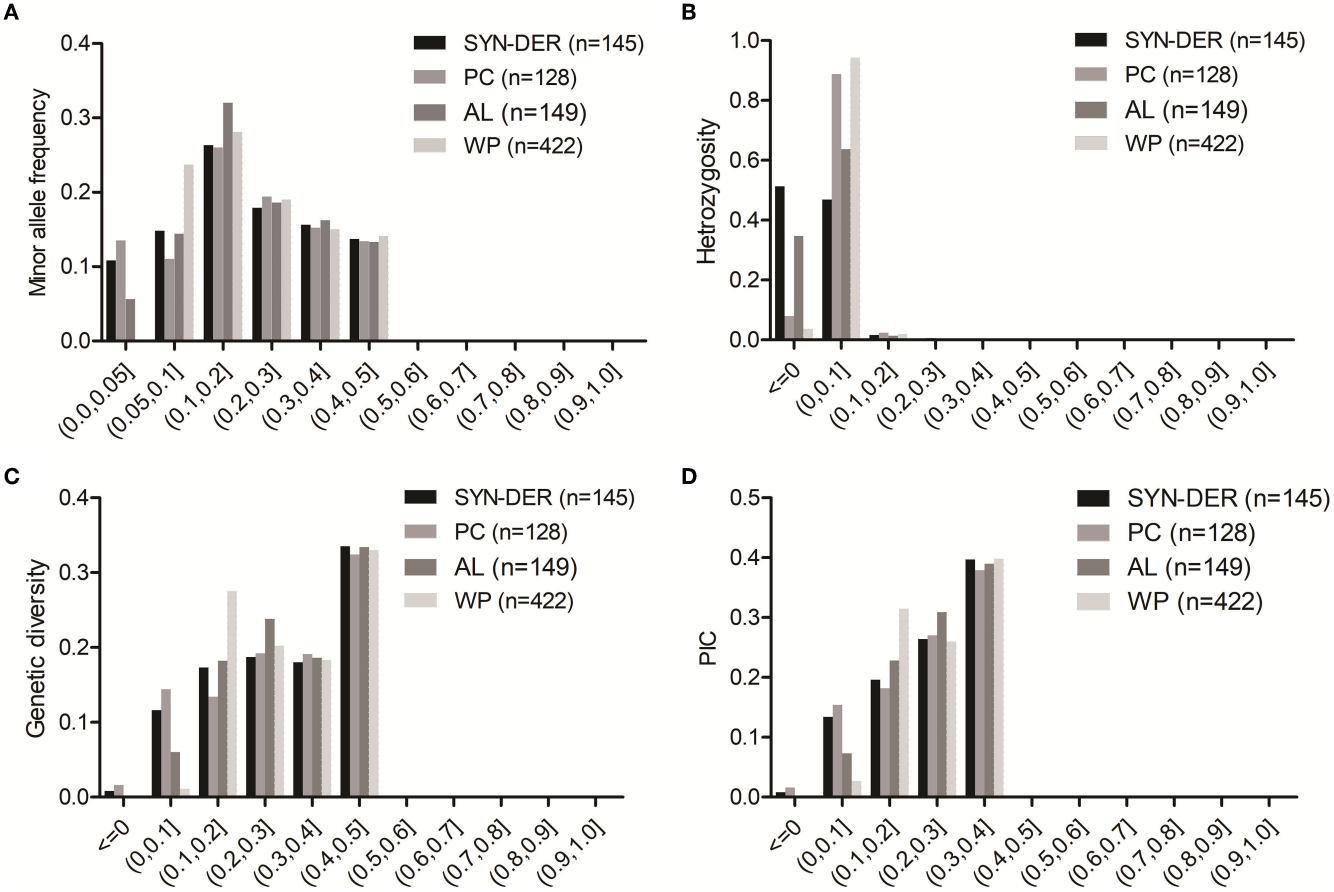
Distribution of minor allele frequency **(A)**, heterozygosity **(B)**, genetic diversity **(C)**, and polymorphic information content **(D)** across 422 accessions (WP), for 145 synthetic-derived wheats (SYN-DER), 128 Pakistan cultivars (PC), and 149 advanced lines (AL) based on 18,589 SNPs.

The genetic diversity parameters including MAF, He, GD, and PIC were calculated using 18,859 GBS-SNPs for the WP and each of the three predefined subpopulations per chromosome in each genome of the panel. The frequency distribution of GBS-SNPs for MAF, He, GD, and PIC is presented in Figure 1. The details of GBS-SNPs per chromosome and across genomes are presented for values of MAF, He, GD, and PIC for WP and subpopulations (Supplementary Table S2). For the WP, as expected the MAF value across genomes ranged from 0.05 to 0.5 with an average of 0.21. As expected, the subpopulations still contained GBS-SNPs with MAF ranging from 0 to 0.05 (Figure 1A). It suggests that some of the common alleles in the WP were rare (MAF < 0.05) in the subpopulations. The PC subpopulation had a higher number of rare GBS-SNPs than the other two subpopulations (i.e., SYN-DER and AL). The numbers of GBS-SNPs with MAF ranged from 0 to 0.05 were 2,015, 2,513, and 1,034 in SYN-DER, PC, and AL subpopulations, respectively (Figure 1A). Viewing the WP, rates of GBS-SNP heterozygosity varied from 0 to 1.99, with an average of 0.019 (Figure 1B). The averaged heterozygosity rate for subpopulations was 0.010 (SYN-DER), 0.036 (PC), and 0.012 (AL) (Supplementary Table S2). The GD values in A, B, and D genomes were 0.319, 0.312, and 0.263, respectively (Supplementary Table S2). At the subpopulation level, SYN-DER (0.294) and AL (0.294) had the highest GD while PC (0.281) had the lowest GD (Supplementary Table S2). The average PIC values varied from 0.09 to 0.375, with an average of 0.245 in the WP (Figure 1D and Supplementary Table S2). The numbers of GBS-SNPs with PIC values ranging from 0.2 to 0.4 were 12,301 (66%), 12,056 (65%), and 12,977 (70%) for SYN-DER, PC, and AL, respectively (Figure 1D).

**Figure 2:**
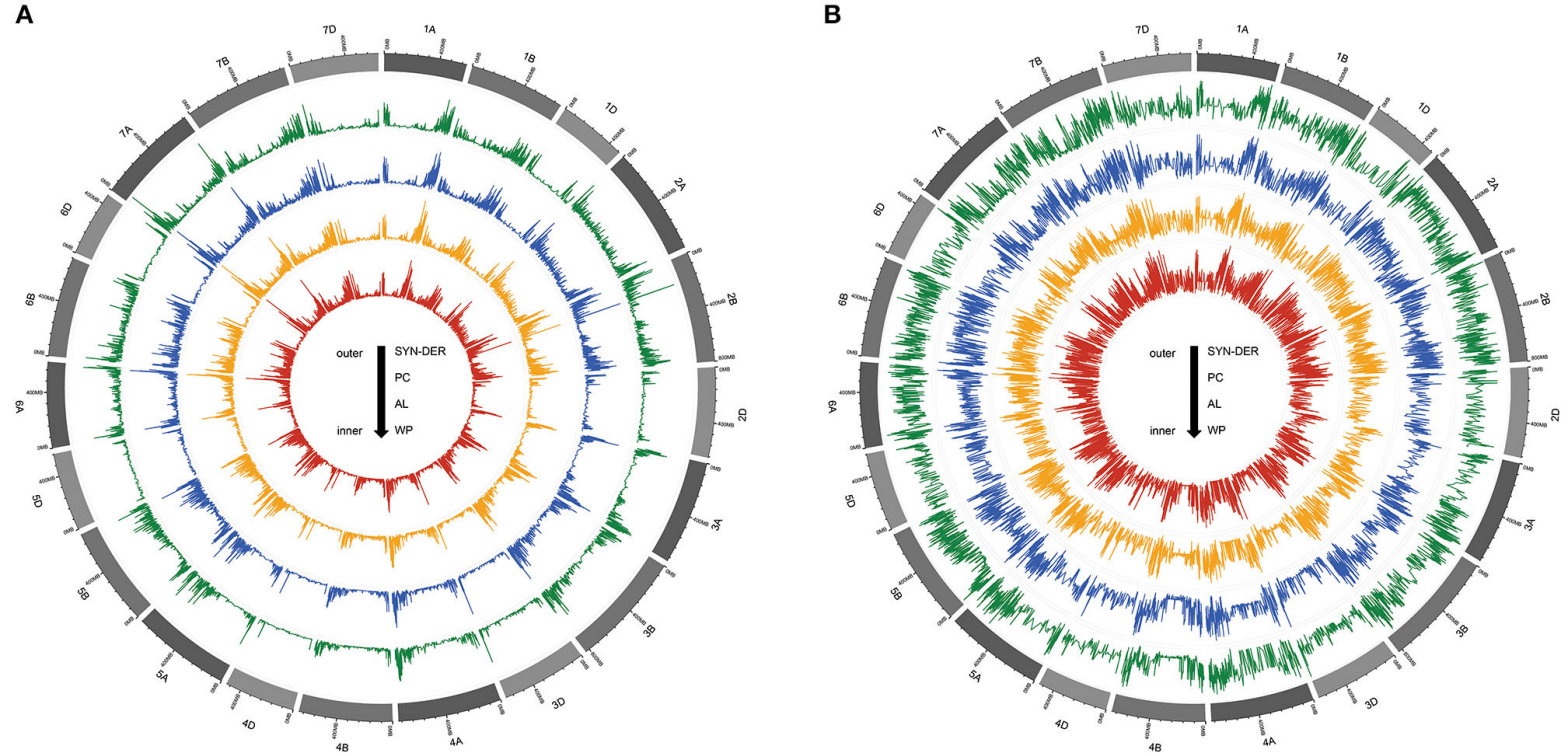
The Circos plot, from outside to inside, illustrates the patterns of nucleotide diversity **(A)** and Tajima'D **(B)** values in synthetic-derived wheats (SYN-DER), commercially released Pakistan cultivars (PC), and advanced breeding lines (AL), respectively. Nucleotide diversity and Tajima'D are plotted along reference chromosomes in sliding windows of 1,000 kb with a step size of 100 kb.

Two types of GBS-SNPs were determined according to nucleotide substitution analysis: (1) transitions (A/G and C/T) and (2) transversions (A/T, A/C, G/T, and C/G). Transition-type SNPs (73.24%) were more frequent than the transversions (26.76%), and transition/transversion (Ts/Tv) ratio was 2.73 (Table 2). The C/T transition type (36.80%) had the highest frequency, while the A/T transversion type (3.67%) had the lowest frequency among all six SNP types of nucleotide substitution (Table 2). The frequencies were almost similar between A/C and G/T. To better understand the patterns of nucleotide variations within a population, we also evaluated the genome-wide nucleotide diversity (also known as π) and Tajima's D statistics based on 18,589 high-quality GBS-SNPs in the WP and among three subpopulations (Figure 2). The mean nucleotide diversity across windows for the WP was estimated at 8.28E-07 (Supplementary Table S3). Nucleotide diversity was high in the telomeric regions than in the pericentromeric regions of all 21 chromosomes (Figure 2A). Furthermore, among the three genomes, D-genome had the lowest nucleotide diversity π and Tajima's D statistics than the A- and B-genomes (Figure 2). The AL (8.32E-07) subpopulation showed the highest nucleotide diversity, followed by SYN-DER (8.12E-07) and PC (7.93E-07) Supplementary Table S3). The average Tajima's D statistics across windows was positive (i.e., 1.42) for the WP (Figure 2B and Supplementary Table S3). On the other hand, the average Tajima's D statistics in SYN-DER, AL, and PC were 1.13, 1.21, and 1.04, respectively. The mean Tajima's D values were positive for the WP and all subpopulations (Supplementary Table S3), reflecting populations may have gone through balancing selection.

**Table 2:** Transition (Ts) and transversion (Tv) SNPs identified using genotyping-by-sequencing.

**SNP type**	**Transtions**		**Transversion**			
	**A/G**	**C/T**	**A/T**	**A/C**	**G/T**	**C/G**
Number of allelic sites	6,773	6,841	683	1,220	1,172	1,900
Frequencies (%)	36.44%	36.80%	3.67%	6.56%	6.30%	10.22%
Total (%)	73.24%	26.76%
TsTv ratio	2.73					

### Population Structure and Genetic Relationships

**Figure 3:**
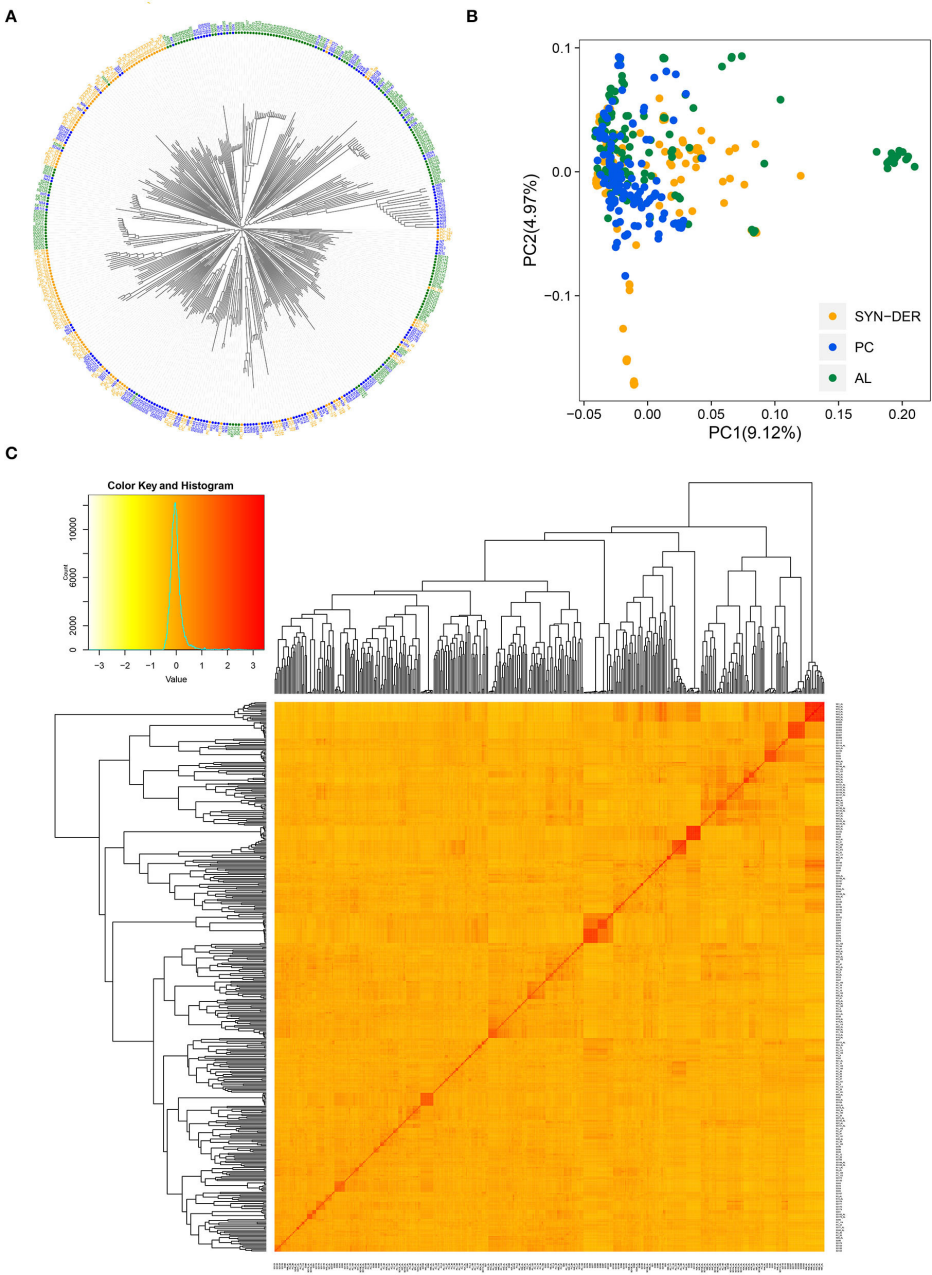
Population structure and diversity analysis of 422 wheat accessions using 18,589 high-quality SNPs. **(A)** Neighbor-joining (NJ) tree, **(B)** principal component analysis (PCA) plot, and **(C)** heat map of pairwise kinship matrix with the tree shown on the top and left. The SYN-DER indicates synthetic-derived wheats; PC, commercially released Pakistan cultivars; AL, advanced breeding lines.

To investigate possible population structure and genetic relationships among the 422 wheat accessions, NJ-tree, PCA, and kinship analyses were conducted (Figure 3). Based on the NJ-tree analysis, we found that the three subpopulations (SYN-DER, PC, and AL) were separated with some admixture (Figure 3A). The AL and SYN-DER were more scattered over PC-1, while PC were more separated along the PC-2. We also inferred the genetic structure and relatedness among the WP by PCA analysis (Figure 3B). In PCA, the first and second principal components explained 9.12 and 4.97% of the total variation, respectively. The weak population structure was detected by both NJ-tree and PCA in the diversity panel, as revealed by Figures 3A,B. The SYN-DER and AL subpopulations were relatively more scattered than the PC subpopulation, indicating that there exists broad genetic divergence in the present collection (Figure 3B). A low level of population structure was also supported by the VanRaden kinship analysis (Figure 3C), which was in accordance with NJ-tree and PCA analyses. The kinship coefficient between pairs of 422 accessions ranged from 0.00 to 3.42 (Figure 3C).

To gain further insights into genetic relationships, we calculated the frequency distribution of kinship coefficients and genetic distances for the WP and the three subpopulations (Supplementary Tables S4, S5). Kinship coefficients near zero indicate no relationship, while those near 2.0 indicate a closer relationship (Supplementary Table S4). For the WP, 60% of the kinship coefficients were equal to 0, 39% varied between 0.01 and 0.8, and the remaining 1% fell between 1.2 and 3.4 (Supplementary Figure S1A). The proportion of kinship coefficients equal to 0 in SYN-DER, PC, and AL were 65, 63, and 60%, respectively (Supplementary Figure S1A). The pairwise genetic distances among the 422 accessions varied from 0 to 0.68 (Supplementary Figure S1B and Supplementary Table S5), with an average of 0.53. The genetic distance between pairs of accessions fell in the range of 0.50 to 0.70 were 82.23% of the WP, 90.06% of the SYN-DER, 90.06% of the PC, and 86.57% of the AL (Supplementary Figure S1B). Most accessions had an estimate between 0.50 and 0.60, regardless of the subpopulations.

Genetic differentiation of predefined subpopulations (i.e., SYN-DER, PC and AL) was assessed using AMOVA analysis (Table 3). AMOVA results showed that 3.41% of the total variation was attributable to the differences among subpopulations, whereas 90.74% was within subpopulations (Table 3). Furthermore, pairwise F_ST_ analysis was computed to investigate subpopulation divergences and presented in Table 4. The F_ST_ coefficient among subpopulations varied from 0.0492 to 0.075. The F_ST_ coefficients showed that the divergence between the SYN-DER and AL (0.0492) was lowest, while the divergence between PC and AL was highest (0.075). Results suggest a low level of genetic differences and in accordance with the NJ-tree and PCA analyses.

**Table 3:** Results from analysis of molecular variance (AMOVA).

**Source of variation**	**df^a^**	**Sum of squares**	**Variance components**	**Variation (%)**
Among subpopulations	2	66796.64	99.49***Va	3.41
Among individuals within population	419	2290148.43	2,647.50***Vb	90.74
Within individuals	422	72048	170.72***Vc	5.85
Total	843	2428993.06	2,917.73	

a *df, degree of freedom*.

“***” The source of variation was highly significant at P ≤ 0.001.

**Table 4:** Pairwise fixation index (F_ST_) between subpopulations SYN-DER (*n* = 145), PC (*n* = 128), and AL (n = 149).

	**SYN-DER**	**PC**	**AL**
SYN-DER	0	***	***
PC	0.0601	0	***
AL	0.0492	0.075	0

*SYN-DER, synthetic-derived wheats; PC, commercially released Pakistan cultivars; AL, advanced breeding lines*.

*** *Represents significant differences between two populations at P ≤ 0.001*.

**Figure 4:**
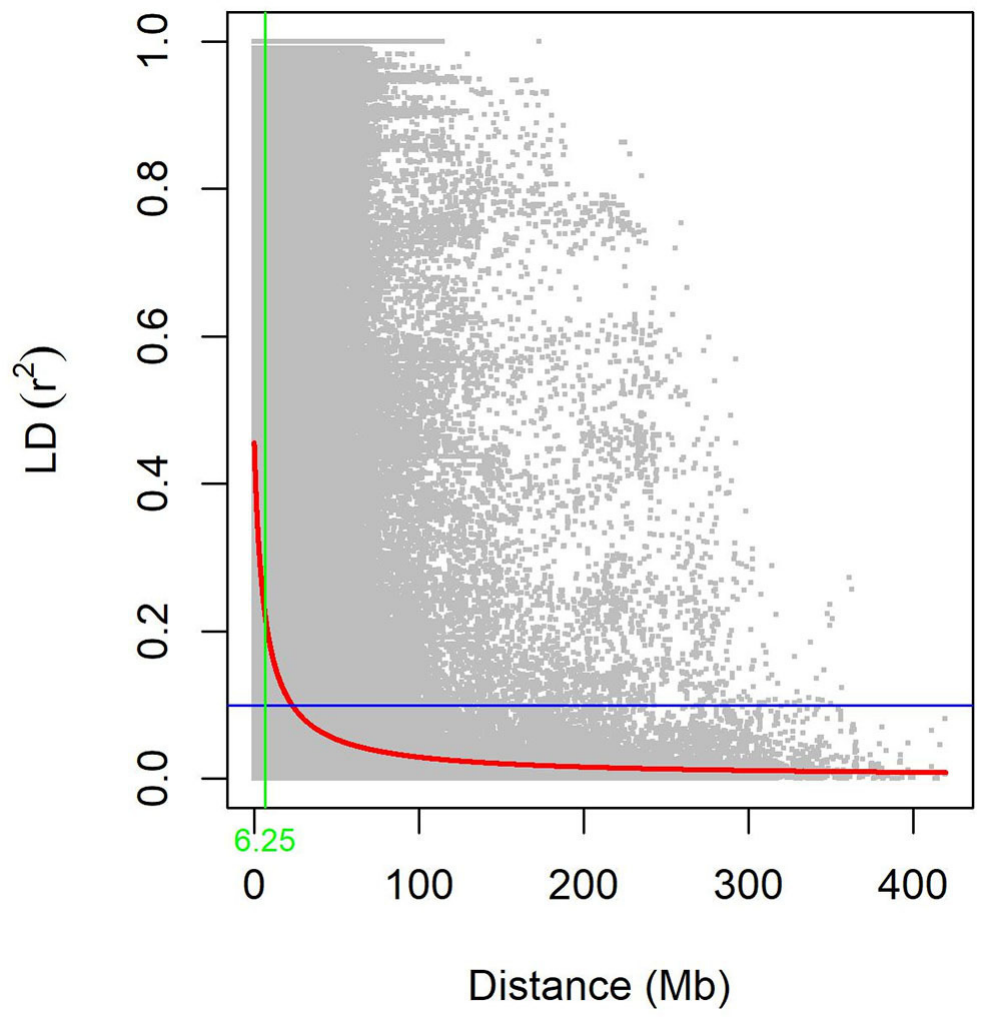
The decay of linkage disequilibrium (LD) in the whole population (WP). Pairwise LD (r^2^) values plotted *vs*. corresponding pairwise physical distance (Mb) of GBS-SNPs. The trend line of non-linear regressions against physical distance is given by the red line. The horizontal and vertical lines represent the critical value of r^2^ (0.1) and LD decay values, respectively.

The summary statistics results for each chromosome in each genome of LD between adjacent GBS-SNPs were computed in the three subpopulations and the WP (Supplementary Table S6). The average *r*^2^ values ranged from 0.06 (5D) to 0.33 (4B). The average *r*^2^ for WP was found to be 0.19. The averaged *r*^2^ reached the lowest in the AL subpopulation (0.06), and the highest in the AL subpopulation (0.35) (Supplementary Table S6). The averaged *r*^2^ was decreased with an increase in distances of the genome for all the subpopulations, suggesting that the probability of LD was low between widely separated SNP pairs (Figure 4 and Supplementary Figure S2). The LD decays at 8.52, 5.79, 8.34, and 6.25 Mb for SYN-DER, PC, AL, and WP at *r*^2^ of 0.1, respectively (Figure 4 and Supplementary Figure S2).

### Identification of Genomic Regions Under Selection by EigenGWAS

**Figure 5:**
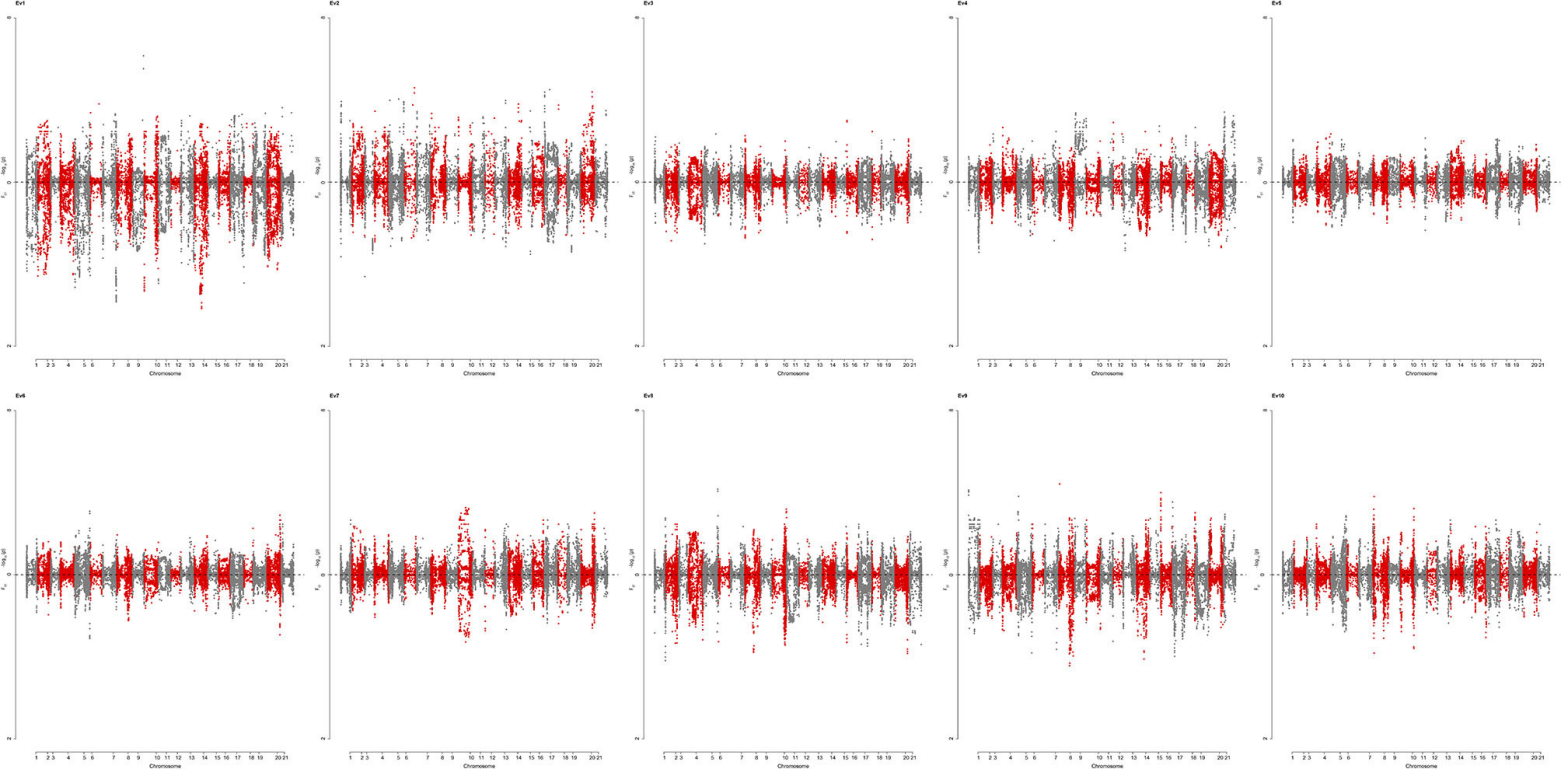
Miami plot showing loci under selection identified by EigenGWAS (upper for PGC and lower for F_ST_) for top 10 eigenvectors (EV) based on 422 wheat accessions. PGC is the *p* corrected by lamdaGC in EigenGWAS. EV1-EV10 were the first 10 eigenvectors, each of which was used as phenotype for EigenGWAS analysis.

To identify genomic regions that have undergone directional selection (the so-called “selection signature”) during wheat improvement, EigenGWAS was conducted based on positive and negative coordinates of the 422 wheat accessions from Pakistan on the corresponding EV, and their selection differentiations were quantified by F_ST_ (Table 5). The average genetic relatedness among 422 Pakistan wheat accessions was −0.0046, suggesting that the effective sample size of the WP was 218.60. The effective number of genome segments was 14.33. The largest eigenvalue was 74.45, while the 10th eigenvalue was 15.61 (Supplementary Table S7). The genomic inflation factor, namely, the λ_GC_ computed from EigenGWAS, which is commonly used in adjusting population stratification for GWAS ranged from 29.98 to 7.20 (Supplementary Table S7). To facilitate the comparisons, EigenGWAS and *F*_ST_ analyses were performed on the WP, and two results were drawn as the Miami plot for each of the 10 EVs (Figure 5). Generally, the peaks from –log_10_(*P*_GC_) and *F*_ST_ fairly mirrored each other, indicating reasonable grouping as defined by *F*_ST_. Overall, EigenGWAS detected selection signatures on all 21 chromosomes (Figure 5 and Supplementary Table S8), while 83 significant GBS-SNPs were identified on 6 of the 10 EVs. To exclude the effect of LD, significant GBS-SNPs overlapping with each other were merged within the 5 Mb genomic window, and highly significant GBS-SNP [i.e., SNP with largest –log_10_(P_GC_)] within one region was used to declare as representative. In total, therefore, 38 selection regions were identified and are shown in Table 5. The total length of the selection regions was 418.97 Mb (Table 5). The distribution selection regions across different chromosomes varied considerably, except for 1B, 1D, 4A, 4B, and 4D. Chromosome 2B (6) and 7B (6) had the highest number of selection regions while 2A (1), 3A (1), 5A (1), 5B (1), 5D (1), 6A (1), 6D, and 7A (1) had the lowest across EVs. Besides, significant selection regions were only identified under EV1 (5), EV2 (24), EV4 (2), EV8 (1), EV9 (5), and EV10 (1) (Table 5). The number of selection regions identified in the B-genome were 19, followed by the D-genome (11), and only 8 selection regions were located in the A-genome. The largest selection region (i.e., 1A_45252081) was identified on chromosome 1A under EV2 spanning roughly over 30 Mb (Table 5). In contrast, a region (i.e., 2B_769950981) on chromosome 2B spanned approximately 5 Mb was the smallest selection region detected under EV8. To understand the biological background of the identified selection regions, we particularly aligned previously reported genes, marker-trait associations, and biparental QTL described for grain yield and yield-related traits, baking quality, disease resistance, adaptation, and flowering-time–related traits (Table 5). Results revealed that 22 (i.e., 57%) out of 38 selection regions were falling within proximity of known functional genes and/or QTL with meaningful agronomic implications as existing support. Results suggest that the 2D region (11.96–21.96 Mb) could be involved in dwarfism in wheat (*Rht8)* and flowering time (*Ppd-D1a.1*) (Table 5). The region 3D (567.09–577.09 Mb) consists of grain color gene *Myb10-D1*, which controls the red pigment of wheat grain. In this region, two QTL for grain yield and kernel width were also reported. The region 1A (0–10.49 Mb) includes one gene and five QTL, which includes a low–molecular weight glutenin subunit *Glu-A3* controlling gluten quality of the wheat, while QTL were associated with phenology-related traits such as grain yield, biological yield, flag leaf length, and kernel width (Table 5). The 5A region spans from 36.22 to 46.22 Mb, which includes photo-period responsive gene (*Ppd-A1)* controlling flowering time in wheat. The 5A region (558.36–569 Mb) consists of QTL associated with spike-related traits in wheat such as spike number, awn length, and spike length (Table 5). Similarly, two regions 5D (436.03–446.03 Mb) and 6B (0–9.52 Mb) also included QTL associated with awn length. A region 7D (42.66 −52.66 Mb) encompasses the *Lr34* that is known to be associated with leaf rust resistance (Table 5). Notably, the association of selection regions with previously known genes/QTL is speculative; however, further pieces of evidence are required to validate the present results. Allele frequencies of selected regions across three subgroups are presented in Supplementary Table S9.

**Table 5:** Top SNPs with a significance of –log_10_ 5.0 between SYN-DER, PC, and AL accessions.

**SNP**	**EV^***a***^**	**CHR^***b***^**	**Region start pos (Mb)**	**Region end pos (Mb)**	**Fst**	**Gene/QTL**	**Trait**	**Reference**
2D_16962948	1	2D	11.96	21.96	0.2259	Rht8, Ppd-D1a.1, QKw + QSpl	Kernel width + Spike length	Tian et al., 2017; Zhou et al., 2018; Li F. et al., 2019
2D_463912950	1	2D	458.91	468.91	0.2402	RHT-8	Plant height,	
3D_572085282	1	3D	567.09	577.09	0.3956	Myb10-D1, QGy + QKw	Seed color, Grain yield + Kernel width,	Li F. et al., 2019
7A_644861968	1	7A	639.86	649.86	0.2869			
7D_53502441	1	7D	48.5	58.5	0.2758	QTkw, GS3-D1	Thousand kernel weight,	Röder et al., 2008
1A_5100794	9	1A	0	10.49	0.0848	Glu-A3, QBy, QHd + Qgy+ QFll + QKw,	Gluten/End-use quality, Biological yield, grain yield + Flag leaf length + kernel width,	Liu et al., 2008; Li F. et al., 2019; Alqudah et al., 2020
1A_45252081	2	1A	17.66	50.83	0.1606	QTkw, QKl + QKns,	Thousand kernel weight, Kernel length + kernel number per spike,	Kumar et al., 2006 + Bhatta et al., 2018; Li F. et al., 2019
2A_41224267	2	2A	36.22	46.22	0.1318	Ppd-A1	Flowering time, QTkw,	Nishida et al., 2013; Bhatta et al., 2018
2B_37251497	2	2B	32.25	42.25	0.1039			
2B_38773853	2	2B	33.77	43.77	0.117			
2B_508214726	2	2B	503.21	513.21	0.169			
2B_775770973	2	2B	770.76	783.47	0.135	QGw	Grain weight,	Alqudah et al., 2020
2D_447834700	2	2D	442.83	452.83	0.1191			
2D_537115124	2	2D	530.56	542.12	0.1295			
3A_20558735	2	3A	15.56	25.56	0.1513	QGy + QBm, QFlw	Grain yield + Biomass weight, Flag leaf width,	Bhatta et al., 2018; Li F. et al., 2019
3B_42330180	2	3B	37.33	47.33	0.1			
5A_564429086	2	5A	558.36	569.43	0.3426	QSn, QAl, QSl + QKw,	SN, Awn length, Spike length+ Kernel width,	Cuthbert et al., 2008; Li F. et al., 2019
5B_527181268	2	5B	515.08	532.18	0.2534	QSpl,	Spike length,	Li F. et al., 2019
5D_441028318	2	5D	436.03	446.03	0.1327	QAl	Awn length	Bhatta et al., 2018
6B_4519965	9	6B	0	9.52	0.1472	QAl, QPh,	Awn length, Plant height,	Bhatta et al., 2018; Li F. et al., 2019
6B_14544540	2	6B	7.74	22.69	0.3018	QFlw	Flag leaf width	Bhatta et al., 2018
6D_24484257	2	6D	19.48	29.96	0.1477	QFlw	Flag leaf width	Bhatta et al., 2018
7B_573733013	2	7B	457.45	467.45	0.225			
7B_579773793	2	7B	568.73	578.73	0.1138			
7B_581047156	2	7B	576.05	586.05	0.1242			
7B_601123999	2	7B	596.12	610.43	0.148			
7B_616469496	2	7B	611.47	621.47	0.1593			
7B_670141587	2	7B	665.14	675.14	0.1037			
7D_559268072	2	7D	554.27	564.27	0.1835	QSn, Qfla	SN, Flag leaf area,	Li et al., 2007; Bhatta et al., 2018
3D_50888526	4	3D	45.63	52.66	0.1203			
7D_47657997	4	7D	42.66	52.66	0.1532	Lr34, QTkw	Leaf rust resistance, Thousand kernel weight	Bhatta et al., 2018
2B_769950981	8	2B	769.95	774.97	0.1948	QGnfs,	Grain number per fertile spikelets,	Alqudah et al., 2020
1A_42100559	2	1A	535.86	545.86	0.1385	QFla, QSdm, QFsps	Flag leaf area + Stem diameter, fertile spikelet per spike	Bhatta et al., 2018; Alqudah et al., 2020
2B_77484831	9	2B	72.48	82.48	0.1472	QTkw, QRl	Thousand kernel weight, root length	Quarrie et al., 2005; Bhatta et al., 2018
3B_40212954	9	3B	35.21	45.21	0.0527			
6A_6562879	9	6A	0	11.56	0.1172	QGw, QPh + QKl,	Grain weight,	Li F. et al., 2019; Alqudah et al., 2020
6B_276518336	2	6B	271.52	281.52	0.1223			
3B_59646004	10	3B	54.65	64.65	0.4	QSd,	Stem diameter,	Bhatta et al., 2018

*Associated traits were obtained around 5 Mb physical nucleotide interval of significant SNP markers of each QTL*.

a *EV, eigenvector*;

b *CHR, Chromosome*.

### Gene Functional Analysis for Selection Regions

**Figure 6:**
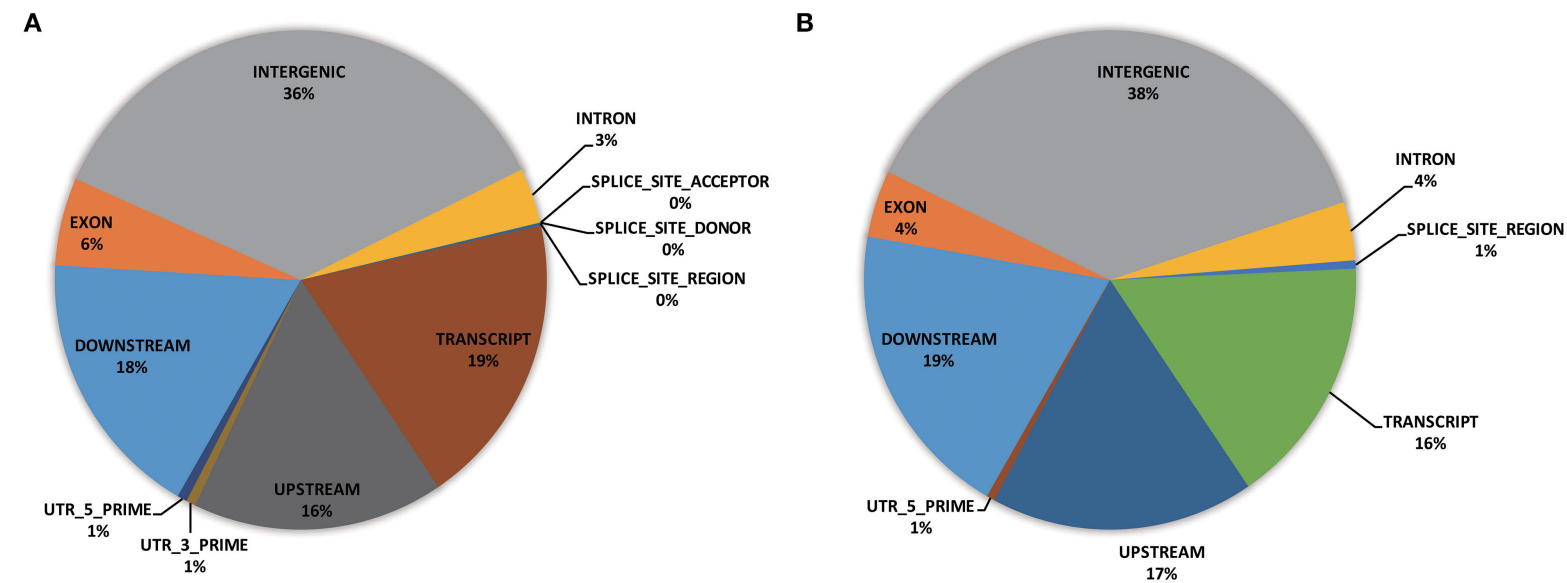
Pie charts depicting genomic annotation for whole-genome GBS-SNPs (18,859 SNPs) **(A)** and significant GBS SNPs identified by EigenGWAS (83 GBS-SNPs) **(B)**.

Functional annotation was carried out to evaluate the genome composition (e.g., intergenic, exon, intron, UTRs) using the whole-genome SNPs (i.e., 18,589 GBS-SNPs) and significant SNPs identified by EigenGWAS (i.e., 38 selection regions) (Figure 6). Of the whole-genome GBS-SNPs, over one-third were located in the intergenic region; more than 15% were in the regions of transcript (i.e., 19%), downstream (18%), and upstream (16%), respectively (Figure 6A). A similar proportion of genome composition could be observed from the gene annotation of 38 selected regions (Figure 6B). Functional enrichment analysis based on genes (IDs recognized by Panther Classification System) within the selected region was performed to identify possible biological pathways associated with the differentially expressed genes (DEGs). Of the 7,263 GO terms annotations, 4,010 GO terms were in the biological function, 2,358 GO terms were in the molecular function, and 895 GO terms were in the cellular component. The distribution of most significantly enriched GO terms revealed several important processes as catalytic activity (GO:0003824), Adenyl ribonucleotide binding (GO:0032559), iron ion binding (GO:0005506), molecular function (GO:0003674), cellular respiration (GO:0045333, GO:0009060), phosphotransferase activity, alcohol group as acceptor (GO:0016773), transferase activity (GO:0016740), ATP binding (GO:0005524), carbohydrate derivative binding (GO:0097367), ribonucleotide binding (GO:0032553), nucleotide binding (GO:0032559, GO:0030554, GO:0032553, GO:0017076, GO:0032555, and GO:0000166), nucleoside binding (GO:1901265 and GO:0035639), NADH dehydrogenase (ubiquinone) activity (GO:0008137), oxidoreductase activity, acting on NAD(P)H (GO:0016651), and so on (Supplementary Figure S3). Significant SNPs identified by EigenGWAS were also subjected to GO enrichment analysis and shown in Supplementary Figure S4. Annotation of DEGs revealed that they were involved in chloroplast organization (GO:0009658), plastid organization (GO:0009657), response to far-red and red light (GO:0010218, GO:0071489, GO:0010017, GO:2000030, GO:0010114), regulation of photomorphogenesis (GO:0010099), lipid modification (GO:0030258), cellular response to light stimulus (GO:0071482), cellular response to radiation (GO:0071478), cellular response to abiotic and environment stimulus (GO:0071214 and GO:0104004), response to salicylic acid and gibberellin (GO:0009751 and GO:00009739), and so on (Supplementary Figure S4).

## Discussion

The hexaploid wheat diversity panel used in the current study was primarily developed in Pakistan and was compared with wheat cultivars from Pakistan used as the reference set. The wheat collection comprised three different subpopulations, which enable us to infer genetic diversity on the basis of high-throughput GBS-SNPs. Consequently, this may allow us to better understand genetic diversity within a germplasm collection to establish genetically divergent heterotic groups, which can be used for wheat improvements in Pakistan. It is generally agreed that subsequent domestication and frequent crossing and selecting among the best genotypes are big challenges for developing high-yielding varieties (Hao et al., 2006; White et al., 2008; Allaby et al., 2019). Introgressing alien chromosomal segments from relative species has its issue referred to as linkage drag of undesirable traits (Klindworth et al., 2013). Novel sources for genetic diversity are expected to be available in less-explored genotypes such as wild relatives, exotic lines, and advanced breeding lines. Furthermore, knowledge about loci that had undergone directional selection is an important step to exploit markers associated with the useful agronomic traits, which might underpin future wheat breeding efforts (e.g., GWAS) as well as to develop *ad hoc* breeding strategies in an attempt to restore part of lost genetic variability (Lopez-Cruz et al., 2015; Taranto et al., 2020).

### Genome-Wide GBS-SNPs Variation

In this study, the 18,589 high-quality GBS-SNPs were identified across the three wheat genomes (A, B, and D) using stringent filtering criteria, and used for downstream analysis (Table 1). In concordance with previous studies based on different types of molecular markers such as GBS-SNPs, 90K SNP array, RFLP, SSR, AFLP, and DArT markers (Liu and Tsunewaki, 1991; Röder et al., 1998; Peng et al., 2000; Chao et al., 2009; Nielsen et al., 2014; Voss-Fels et al., 2015; Alipour et al., 2017; Eltaher et al., 2018), we found that a high level of GBS-SNPs were located in the B-genome, while low levels were located in the D-genome, indicating that D-genome is the least diverse wheat genome. Furthermore, Dubcovsky and Dvorak (2007) concluded that a large proportion of natural gene diversity in hexaploid wheat came from the polyploid nature of its tetraploid ancestor (AABB) than the diversity found in *Ae. tauschii* (DD) during domestication. This conclusion could be a good explanation of the high levels of GBS-SNPs in the A- and B-genomes observed in this study (Table 1). The filtered markers spanned a physical distance of 14,053.07 Mb, with an average marker density of 1.28 Mb/marker for the WP, which was slightly lower than previous reports on wheat (Liu et al., 2019). The present study observed more transitions-type SNPs than transversion-type SNPs across three wheat genomes (Table 2), which is in agreement with several previous studies on hexaploid wheat (Alipour et al., 2017; Kumar et al., 2020). The abundance of the transition-type SNPs was due to the mutation of methylcytosine to uracil and then into thymine (Alipour et al., 2017). The hexaploid wheat genome is highly methylated because it arose from two polyploidization events, which may explain transition-type SNP abundance in wheat. Moreover, various studies support the fact that transition-type SNPs are preferred over transversion-type SNPs, in addition to InDels or multiple allelic SNPs for SNP array development (Bianco et al., 2016; Clarke et al., 2016). The higher Ts/Tv ratio improves the accuracy of SNP prediction with a greater level of confidence.

### Estimation of Genetic Diversity

The molecular characterization of genetic resources remains the most promising option for efficient conservation and sustainable use of their diversity in crop breeding (Alipour et al., 2017; Liu et al., 2019). Genetic variability in Pakistan wheat panel revealed by GD and PIC reflected genetic diversity at the nucleotide level of a genetic population which is a key to understanding the effect of past selective forces on germplasm resources. The average GD (0.30) and PIC (0.25) were estimated in the WP in this study, which is fairly similar to GD and PIC values in previous investigations on wheat (Eltaher et al., 2018; Mourad et al., 2020). On the other hand, Kumar et al. (2020) reported higher GD and PIC values for a set of 483 spring wheat genotypes from India genotyped with 35K Axiom Wheat Breeder's Array. In the present study, AL and SYN-DER subpopulations had higher GD than the PC subpopulation, possibly reflecting recent breeding progress in the diversification of germplasm resources (Supplementary Table S2). Similarly, the higher PIC value was also noted for the AL subpopulation, followed by SYN-DER and PC subpopulations (Supplementary Table S2). Moreover, considerable variation was also noted within the different subpopulations for diversity among the three wheat genomes. As expected, the D-genome showed the lowest genetic diversity for all three subpopulations (Liu et al., 2019). These observations were further supported by nucleotide diversity π and Tajima's D analysis (Figure 2). The differences in genetic diversity among AL, SYN-DER, and PC subpopulations indicated that AL and SYN-DER subpopulations were relatively more diverse. This might be because AL and SYN-DERs subpopulations were developed by crossing Pakistan and exotic parental genotypes as parents (Supplementary Table S1), and have been selected in the field for agronomic superiority (Afzal et al., 2019). It is also possible that artificial selection has fixed targeted regions and resulted in genetically homogenous individuals. Consequently, the genotypes present in AL and SYN-DER subpopulations can be used to enhance genetic variation for selection and to accelerate wheat improvement.

### Genetic Structure and Relatedness

The main challenges associated with the analysis of any genetic data are (1) to explore whether the studied population is genetically homogeneous or contains distinct subgroups, and (2) to find quantitative evidence that supports the presence of these subgroups (Patterson et al., 2006). In this study, NJ-tree, PCA, and pairwise kinship analyses were used to assess the population structure of 422 wheat accessions (Figure 3). Interestingly, these different analyses could not differentiate accessions from predefined subpopulations, which raises the possibility of exchanging adapted germplasm in crop improvement activities within the country. It has been widely reported that foreign wheat genotypes (e.g., Mexico and United States) have extensively been used as parents in Pakistan crossbreeding programs that lead to new cultivars (Ain et al., 2015; Rasheed et al., 2016; Liu et al., 2019), which was in general agreement with pedigree information (Supplementary Table S1). Furthermore, the targets of Pakistan crossbreeding programs included improvement of yield potential; resistance leaf and stripe rust; and tolerance to drought, salinity, and terminal heat stress (Rasheed et al., 2016), which could be another reason for overlap between accession from predefined subpopulations. The present results are generally consistent with several other studies (Rasheed et al., 2016; Afzal et al., 2019) which reported overlap between bread wheat cultivars (or landraces) and synthetic derivatives based on genotypic data. However, it was also noted that advanced lines derived from synthetic wheat were separated from non-synthetic wheat.

The resolution in terms of similarity, the coefficient of kinship matrix is dependent on the number of genotypes and markers used in a study. The low numbers will restrict the exploitation purposes in deciphering novel alleles for economic traits and will exhaust in the short term. Large numbers of both genotypes and markers will increase the dissimilarity coefficients, and this can give a possible overview of the collection in use. It is recommended for long-term breeding goals to explore genetic relatedness and divergence among genotypes and to subject for high-density genotyping (Kumar et al., 2020). Regarding kinship, 60% of the pairwise kinship estimates were equal to zero, indicating that these accessions were distantly related (Supplementary Figure S1A). The kinship estimates identified in the present study could be useful to avoid inbreeding. The average Rogers' genetic distance was larger for SYN-DER as compared with AL and PC (Supplementary Table S4). Approximately, 82% of pairwise comparisons of genetic distance among 422 accessions were in the range of 0.50–0.70 (Supplementary Figure S1B). Consequently, these results provide evidence of a very low degree of genetic redundancy with this diversity panel and support our conclusion that the AL and SYN-DER subpopulations are genetically diverse (Figure 3). Furthermore, AMOVA suggested a high degree of genetic diversity within subpopulations and a low degree of variation among populations (Table 3). These variations were highly significant according to the partition value (*p* < 0.001). The selection for agronomic traits in the Pakistan crossbreeding programs was considered the main reason for this high variation within subpopulations. The low degree of diversity among subpopulations could be due to high genetic exchange or gene flow (Eltaher et al., 2018; Kumar et al., 2020). Therefore, breeders can select genotypes as parents in crossbreeding for improving economic traits, from the same subpopulation than selecting from different subpopulations. Pairwise *F*_ST_ among subpopulations revealed moderate genetic differentiation (Table 4), which is in agreement with population structure analysis (Figure 3). In the present study, the low level of *F*_ST_ was found between AL and SD, indicating a low-level genetic differentiation between these two subpopulations (Table 4). This coincided with the AMOVA result (Table 3), where a large proportion of genetic diversity was accounted for within subpopulations. LD reflects the degree of linkage between loci, referring to the non-random association of two or more loci in the genome, and influences the genetic forces that structure a population (Morrell et al., 2012). LD decay is one of the most important factors in evaluating the marker coverage to determine the resolution of GWAS results. It is well reported that different populations and different genomic regions of chromosomes always show varied LD, in accordance with the results of the present study and with previous reports (Morrell et al., 2012; Afzal et al., 2019; Liu et al., 2019). In the present collection, the overall genome-wide LD decay was shorter than that reported for other investigations on Pakistan wheat germplasms using different classes of molecular markers (Afzal et al., 2019; Liu et al., 2019). Some researchers have reported low or null decay in diversity in different germplasm resources (e.g., landraces and modern cultivars), although they observed the impact of breeding on LD patterns and allele frequency (Taranto et al., 2020). The distances of LD decays in the SYN-DERs and AL were higher than in the PC due to SYN-DERs and AL germplasm under high directional selection pressure than in the PC. In all three subgroups, the mean r^2^ value was higher than for the entire population (Supplementary Table S6), indicating that more alleles are in LD with a weak population genetic structure.

### Implications for Wheat Breeding

The genetic bottleneck is an important challenge in crop breeding and artificial selection, which also eliminates standing variation of surrounding genomic regions. Identification of genomic regions for artificial selection is a basic step in understanding breeding history (Liu et al., 2019; Li J. et al., 2020). The trait-associated genes/QTL identified within selection regions should have undergone selection during wheat breeding activities and could be valuable for marker-assisted selection of traits useful for agriculture and assist the use of germplasm. In the present study, 38 genomic regions were found in the present diversity panel and were compared with previously known genes and reported QTL in different wheat populations (Table 5). From population structure analysis, there is no clear separation among the three subpopulations (Figure 3), and in agreement with pairwise F_ST_ calculated among the three subpopulations (Table 4). Therefore, EigenGWAS approach was chosen because it does not require predefining the subpopulations (Chen et al., 2016). An eigenvalue reflects the mean genetic variation captured and was used as the phenotype in EigenGWAS. Whereas λ_*GC*_ of eigenvector in EigenGWAS represents median of variation (Supplementary Table S6). Moreover, the difference between eigenvalue and λ_*GC*_ is equivalent to the difference between the mean and a median of a population, implicating the existence of strong selection that could be due to natural or artificial selection during domestication or breeding (Li J. et al., 2019; Liu et al., 2019). In recent studies, the EigenGWAS combined with *F*_ST_ analysis has been deployed to identify wheat selection regions (Afzal et al., 2019; Liu et al., 2019). Liu et al. (2019) detected genomic regions by wheat 90 K SNP array in 687 accessions, mainly collected from Pakistan and China, and found that most of the selected regions were associated with known phenotypes for disease resistance, vernalization, quality, and adaptability traits. Afzal et al. (2019) investigated 240 Pakistan wheat accessions, including 171 accessions for SYN-DERs and 69 accessions for PC and AL with wheat 90 K SNP array, and found 89 selection regions within the proximity of functional genes associated with phenology-related traits such as *Vrn-D3* and *TaElf3-D1* for flowering time, *TaCwi-A1, TaCKX-D1, TaSus1-7A*, and *TaGS-D1* for grain size and weight. However, the use of 90K SNP array could lead to ascertainment bias because the representative SNPs were discovered mainly from the wheat cultivars from Australia, United States, and Europe. The majority of selected regions identified in the present diversity panel were located in B genome (50%) as compared to D-genome (29%) and A-genome (21%), a finding consistent with previous reports (Afzal et al., 2019; Liu et al., 2019), reflecting that the B-genome has experienced intense selection pressure than the D-genome. A total of 38 selected regions were identified in the present study. Of which 22 selected regions were overlapped with previously reported functional genes or/and QTL for important agronomic traits including yield-related traits (QGy, QGw, QTkw, QKw, QSn, QKl), plant height (*Rht-8*, QPh), end-use quality (*Glu-A3*), flag-leaf-related traits (QFla, QFlw, QFll), biotic resistance (*Lr34*), and vernalization (*Vrn-D3* and *Ppd-A1*) (trait nomenclature is presented in Table 5 legends; Table 5). Similar investigations reported in other crops such as maize, barley, and soybean, also suggested that most of the selected regions are associated with phenology-related traits (Liu et al., 2017; Li J. et al., 2019, 2020; Li Z. et al., 2020). Our findings suggest that the selected regions observed in the Pakistan diversity panel may be (or have been) under direct selection and are plausible because it reflects wheat breeding targets in Pakistan. These selected regions will be of interest to further understand their contribution to crop improvement and adaptation of Pakistan wheat germplasm resources.

Although many selection regions had been identified in Pakistan wheat germplasm before (Afzal et al., 2019; Liu et al., 2019), the function of many genomic regions remains unclear. Thus, it is crucial to gain more information on the architecture of selected regions. The functional annotation results revealed that most of the loci were mapped to intergenic regions than that to coding regions (Figure 6), in agreement with previous reports (Jordan et al., 2015). Several studies reported that intergenic regions are genetically diverse and are associated with phenotypic variations (Mei et al., 2018). Viewing the whole-genome GBS-SNPs, the GO analysis revealed that inferred genes were mainly associated with molecular functions (e.g., catalytic and enzymatic activity), the biological process of protein phosphorylation, cellular respiration, aerobic respiration, signal transduction, and cellular component (e.g., Photosystem II reaction center) (Supplementary Figure S1). Whereas, annotation results of selected regions showed inferred genes mostly encoding chloroplast and plastid organization, lipid oxidation, cellular response to red or far-red light, cellular response to abiotic, environment stimulus, response to gibberellin, response to salicylic acid, and so on (Supplementary Figure S2). For instance, response to salicylic acid (GO:0009751) was a significant GO term, which controls the growth and stress response (e.g., drought) in wheat (Loutfy et al., 2012). Liu et al. (2019) identified drought tolerance genes (*NAM-6A*, and *1-FEH-w3*) within the selection regions in Pakistan wheat germplasm, which supports our observation. Comprehensive knowledge of genetic diversity, population structures, and the identification of selection regions offer the potential to assist plant breeders in better understanding the implications of the selection regions on targeted crop improvement and facilitate the use of germplasm.

## Data Availability Statement

The datasets presented in this study can be found in online repositories. The names of the repository/repositories and accession number(s) can be found below: https://datadryad.org/stash/share/ts92LqrXBJVsNhZEd8punew3Uv6irdDkdBuHxC4V4IQ.

## Author Contributions

HL conceived and designed the experiments. AR performed NGS bioinformatics and genotyping. MA conducted the experiments and wrote the manuscript under the supervision of HL. MA, SD, and HS performed statistical analyses. HL and AR revised the manuscript. JW and ZH reviewed the manuscript. All authors read the final version of the manuscript and approved it for publication.

## Funding

This work was supported by the project of Hainan Yazhou Bay Seed Lab (B21HJ0223) and the National Science Foundation of China (32022064).

## Conflict of Interest

The authors declare that the research was conducted in the absence of any commercial or financial relationships that could be construed as a potential conflict of interest.

## Publisher's Note

All claims expressed in this article are solely those of the authors and do not necessarily represent those of their affiliated organizations, or those of the publisher, the editors and the reviewers. Any product that may be evaluated in this article, or claim that may be made by its manufacturer, is not guaranteed or endorsed by the publisher.

## Supplementary Material

Supplementary Figure S1 Distribution of pairwise kinship values **(A)** and Roger's genetic distances **(B)**. The SYN-DER indicates synthetic-derived wheats; PC, commercially released Pakistan cultivars; AL, advanced breeding lines; WP, all 422 wheat accessions.

Supplementary Figure S2 The decay of linkage disequilibrium (LD) in the synthetic-derived wheats **(A)**, commercially released Pakistan cultivars **(B)**, advanced breeding lines **(C)**. Pairwise LD (r^2^) values plotted vs. corresponding pairwise physical distance (base pairs) of GBS-SNPs. The trend line of non-linear regressions against physical distance is given by the red line. The horizontal and vertical lines represent the critical value of r^2^ (0.1) and LD decay values, respectively.

Supplementary Figure S3 Gene ontology (GO) enrichment analysis for the whole population. Top 30 significantly enriched GO −log_10_ (p) terms irrespective of biological processes, cellular components, and molecular functions.

Supplementary Figure S4 Gene ontology (GO) enrichment analysis for selected regions identified by EigenGWAS. Top 30 significantly enriched GO −log_10_ (p) terms irrespective of biological processes, cellular components, and molecular functions.

Supplementary Table S1 Distribution of the 422 wheat accessions.

Supplementary Table S2 The averaged genetic diversity parameters across chromosomes and genomes for each subpopulation (a–c). The SYN-DER indicates synthetic-derived wheats; PC, commercially released Pakistan cultivars; AL, advanced breeding lines.

Supplementary Table S3 The nucleotide diversity (pi) and Tajima'D summary statistics in the whole population (WP), synthetic-derived wheats (SYN-DER), commercially released Pakistan cultivars (PC), and advanced breeding lines.

Supplementary Table S4 The relative kinship between pairs of 422 accessions computed from 18,589 GBS-SNPs. The SD indicates synthetic-derived wheats; PC, commercially released Pakistan cultivars; AL, advanced breeding lines.

Supplementary Table S5 Rogers' genetic distance among pairs of 422 accessions calculated from 18,589 GBS-SNPs. The SD indicates synthetic-derived wheats; PC, commercially released Pakistan cultivars; AL, advanced breeding lines.

Supplementary Table S6 The average r^2^ values of each pairwise SNPs for each chromosome and genome. The SYN-DER indicates synthetic-derived wheats; PC, commercially released Pakistan cultivars; AL, advanced breeding lines.

Supplementary Table S7 Summary statistics from EigenGWAS analysis for 422 wheat accessions.

Supplementary Table S8 Significant SNPs detected by EigenGWAS under the cutoff of −log_10_ (PGC = 5.00).

Supplementary Table S9 Allele frequency of the SNPs within selection loci across three germplasm groups.
