# Supplementary figures and images for: Genetic Diversity and Selection Signatures in Synthetic-Derived Wheats and Modern Spring Wheat
Source: Front Plant Sci. 2022 Jul 12;13:877496. doi: 10.3389/fpls.2022.877496 (PMC9315363; doi:10.3389/fpls.2022.877496)

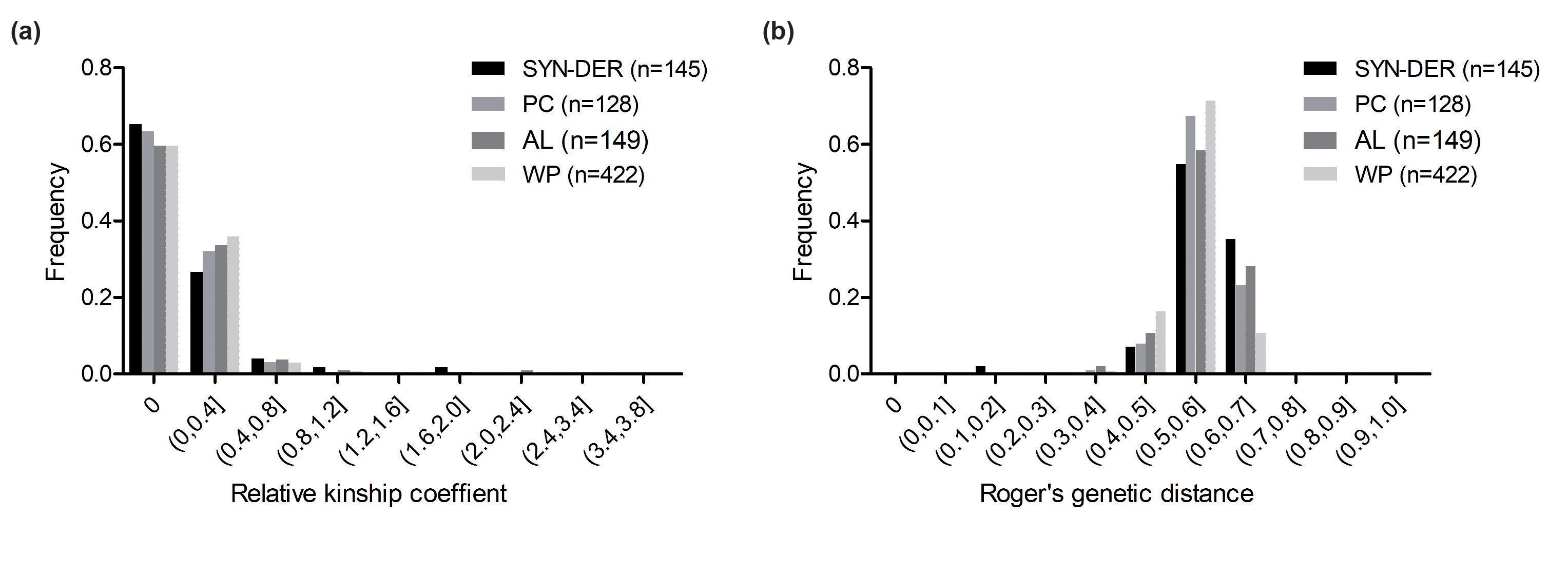

Supplement: Supplementary Figure S1 — Distribution of pairwise kinship values (A) and Roger's genetic distances (B). The SYN-DER indicates synthetic-derived wheats; PC, commercially released Pakistan cultivars; AL, advanced breeding lines; WP, all 422 wheat accessions. [file Image_1.jpg]

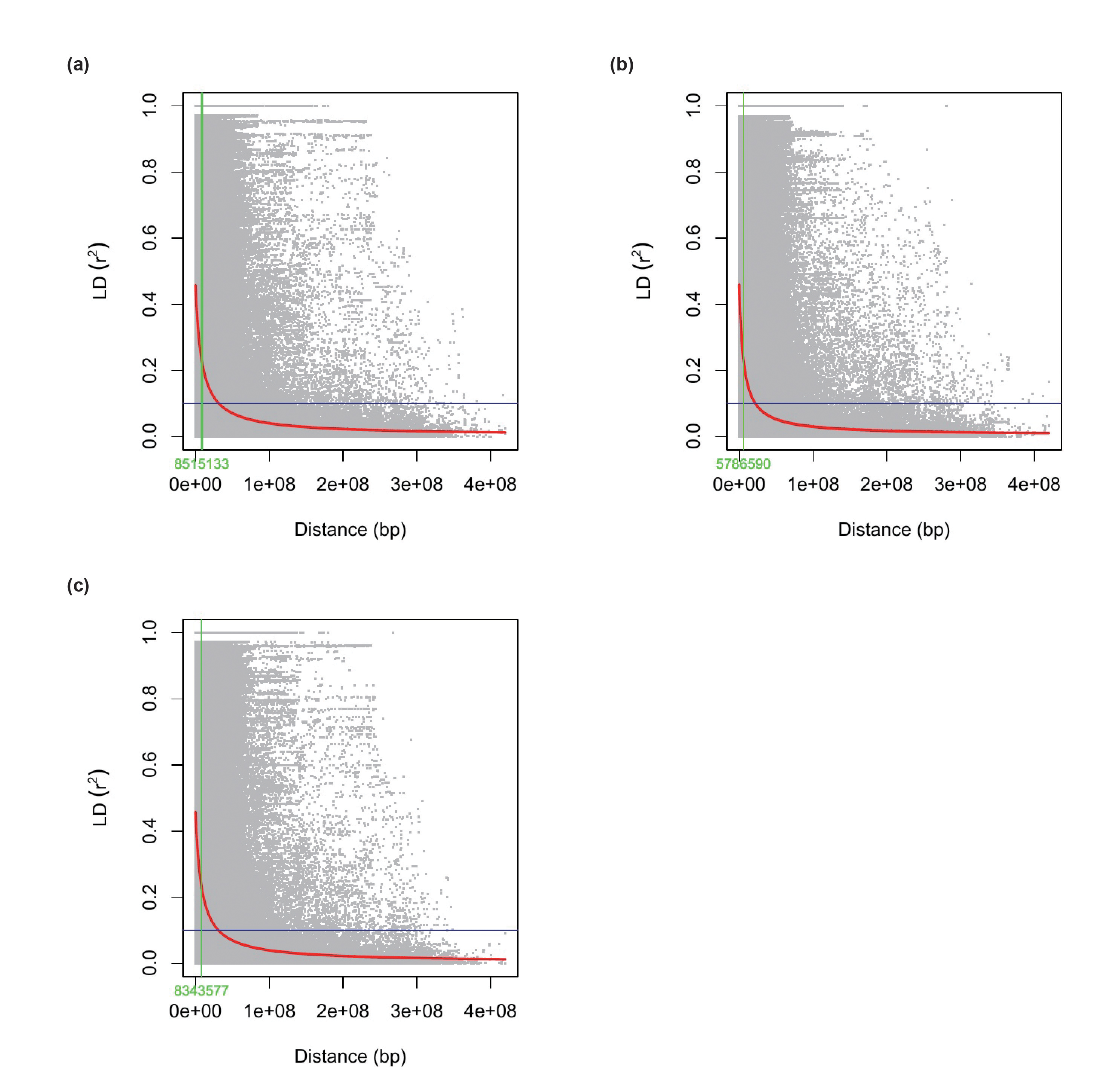

Supplement: Supplementary Figure S2 — The decay of linkage disequilibrium (LD) in the synthetic-derived wheats (A), commercially released Pakistan cultivars (B), advanced breeding lines (C). Pairwise LD (r2) values plotted vs. corresponding pairwise physical distance (base pairs) of GBS-SNPs. The trend line of non-linear regressions against physical distance is given by the red line. The horizontal and vertical lines represent the critical value of r2 (0.1) and LD decay values, respectively. [file Image_2.jpg]

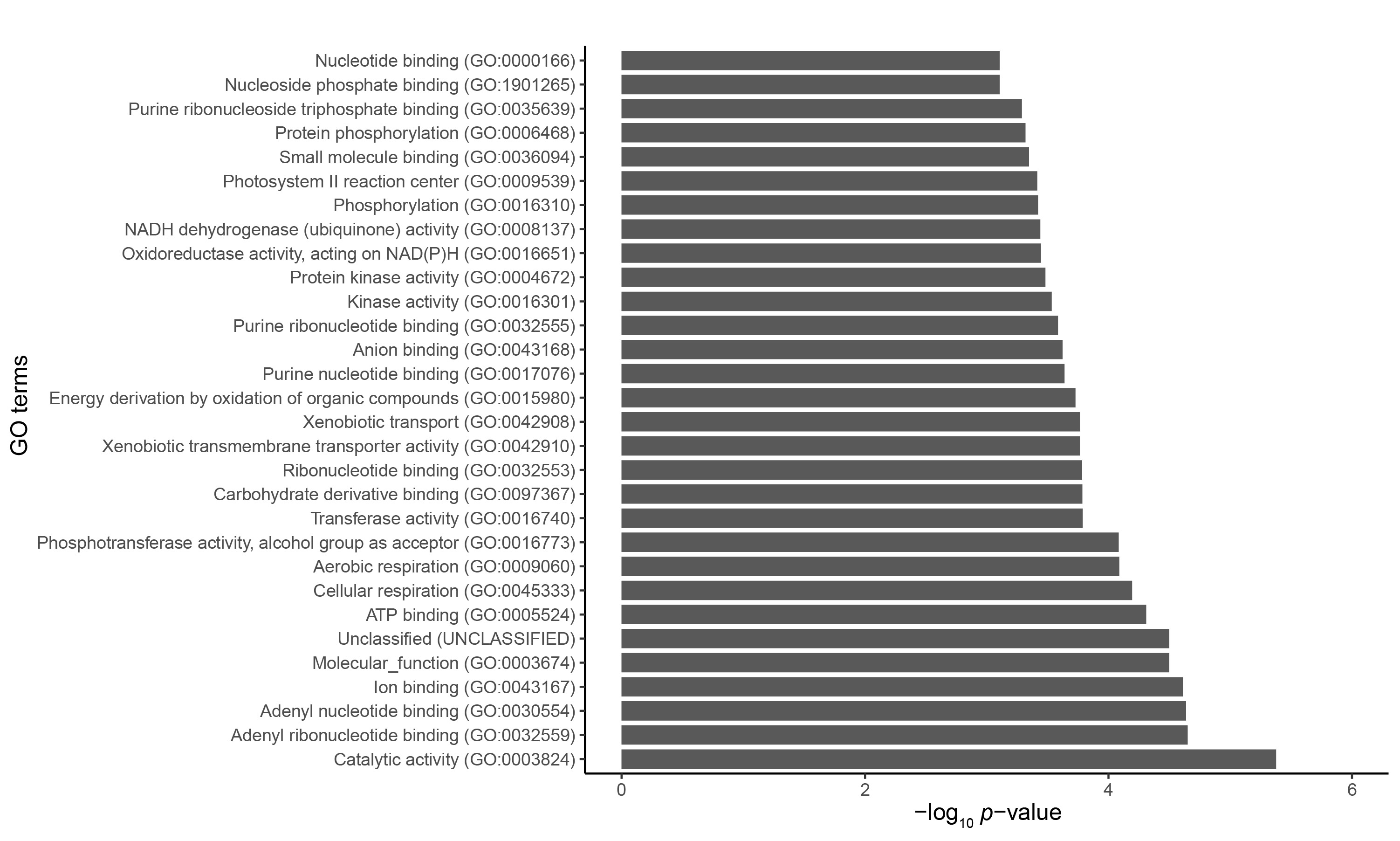

Supplement: Supplementary Figure S3 — Gene ontology (GO) enrichment analysis for the whole population. Top 30 significantly enriched GO −log10 (p) terms irrespective of biological processes, cellular components, and molecular functions. [file Image_3.jpg]

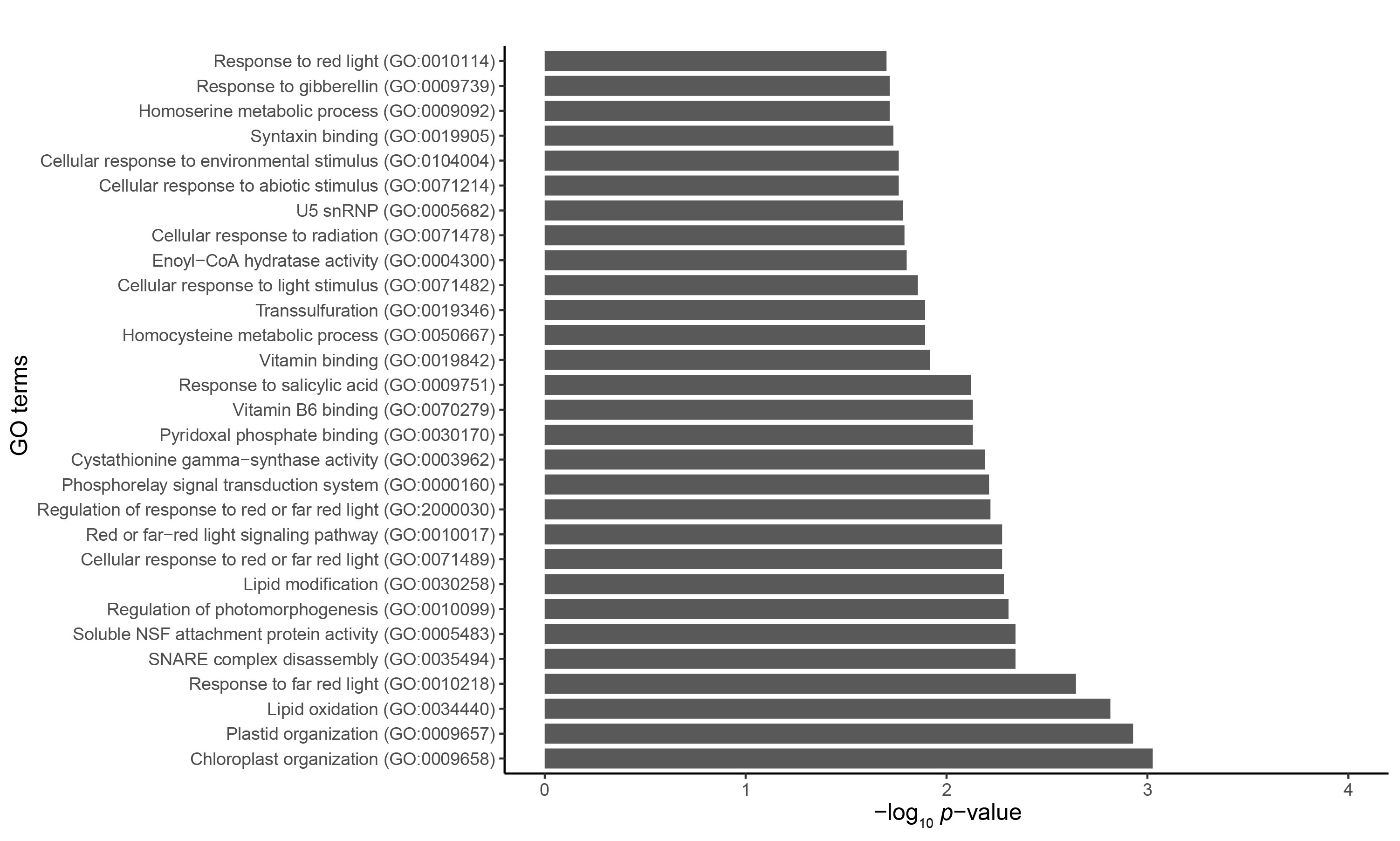

Supplement: Supplementary Figure S4 — Gene ontology (GO) enrichment analysis for selected regions identified by EigenGWAS. Top 30 significantly enriched GO −log10 (p) terms irrespective of biological processes, cellular components, and molecular functions. [file Image_4.jpg]
